# Pathophysiology and Symptoms of Renal Colic in Children – a Case Report

**DOI:** 10.34763/devperiodmed.20182203.265269

**Published:** 2018-10-04

**Authors:** Magda Rakowska, Katarzyna Królikowska, Katarzyna Jobs, Małgorzata Placzyńska, Bolesław Kalicki

**Affiliations:** 1 Department of Paediatrics, Nephrology and Allergology Military Institute of Medicine, Warsaw, Poland

**Keywords:** renal colic, pain, urolithiasis, children, pathophysiology, treatment, kolka nerkowa, ból, kamica nerkowa, dzieci, patofizjologia, leczenie

## Abstract

Urolithiasis is a disease characterized by the presence of stones in the kidney or urinary tract. It is often detected accidentally during an ultrasound or an abdominal x-ray performed for other reasons. However, the first symptom of kidney stone disease can be severe pain called renal colic. Pain caused by a colic attack is characterized by sudden onset. In half of the cases it is associated with nausea or vomiting and can lead to hypotension and fainting. The exact location and radiation of the pain depends on the location of the stone in the urinary tract. The first most commonly performed study is abdominal ultrasound with estimation of the deposit size and evaluation of urinary tract obstruction. Alternative or complementary studies are: an abdominal x-ray where radiopaque deposits can be shown, or unenhanced helical computed tomography of the abdomen. The severity of pain depends on the individual pain threshold and on the change in hydrostatic pressure in the part of the urinary system above the obstruction. Prolonged deposition of the stone in one place causes the activation of autoregulatory mechanisms to lower the pressure of the upper urinary tract, which limits the pain. The basic treatment for renal colic is analgetic therapy. The most commonly used drugs are NSAIDs and opiates. Another important component of renal colic treatment are medications that facilitate urinary stone passage by reducing oedema or limiting urethral contractions, such as: calcium channel blockers, alpha blockers, phosphodiesterase inhibitors. Intensive hydration is not currently recommended. Patients who are unlikely to spontaneously excrete the stone are eligible for minimally invasive treatment. The risk of urolithiasis recurring is high, reaching up to 40% in 5 years and up to 50% in 10 years. However, it can be reduced by proper prevention. The paper describes the pathophysiology of pain in renal colic, the treatment methods, and the case of a boy with recurrent renal colic.

## Streszczenie

Kamica układu moczowego jest chorobą, którą charakteryzuje obecność złogów w nerkach lub w drogach moczowych. Jest ona często wykrywana przypadkowo podczas badania USG lub RTG jamy brzusznej wykonywanych z innych przyczyn. Jednak pierwszym objawem kamicy może być ostry ból noszący nazwę „kolki nerkowej”. Ból spowodowany napadem kolki jest to ból o nagłym początku, w połowie przypadków towarzyszą mu nudności lub wymioty, może dojść do hipotensji i omdlenia. Dokładne umiejscowienie i promieniowania bólu zależy od lokalizacji złogu w drogach moczowych. Najczęściej pierwszym wykonywanym badaniem jest USG jamy brzusznej z oceną wielkości złogu oraz stopniem blokady odpływu moczu. Alternatywą lub badaniem uzupełniającym jest RTG przeglądowe jamy brzusznej, na którym zostaną uwidocznione złogi nieprzepuszczalne dla promieni rentgenowskich lub niskodawkowa spiralna tomografia komputerowa jamy brzusznej bez środka kontrastowego. Nasilenie dolegliwości bólowych zależy od indywidualnego progu bólowego oraz od szybkości zmian ciśnienia hydrostatycznego w części układu moczowego powyżej przeszkody. Przedłużone przebywanie złogu w jednym miejscu daje warunki do uruchomienia mechanizmów autoregulacyjnych mających na celu obniżenie ciśnienia w górnej części dróg moczowych, co ogranicza dolegliwości bólowe. Podstawą leczenia kolki nerkowej jest leczenie przeciwbólowe. Najczęściej stosowanymi w tym celu lekami są NLPZ oraz opioidy. Kolejną ważną składową leczenia kolki nerkowej stanowią leki ułatwiające wydalanie złogu, działające głównie poprzez redukcję obrzęku lub ograniczenie skurczu moczowodu, takie jak: blokery kanału wapniowego, alfa blokery, inhibitory enzymu fosfodiesterazy. Aktualnie nie zaleca się intensywnego nawadniania pacjenta. Chorych, u których mało prawdopodobne jest samoistne wydalenie złogu, kwalifikuje się do małoinwazyjnego leczenia zabiegowego. Ryzyko nawrotów kamicy układu moczowego jest duże, sięga do 40% w ciągu 5 lat oraz do 50% w ciągu 10 lat, może być jednak zredukowane poprzez stosowanie właściwej profilaktyki. W pracy opisano patofizjologię bólu w kolce nerkowej, metody leczenia, a także przypadek chłopca z nawracającą kolką nerkową.

## Symptoms of renal colic

Urolithiasis is a disease characterized by the presence of stones in the kidney or urinary tract. It is most frequently diagnosed between 20 and 60 years of age [1], but also affects pediatric patients. The majority of children with urinary stones present a metabolic tendency to develop kidney calculi, such as hypercalciuria [2, 3]. The most common substances in the deposits are: calcium, oxalates, uric acid, magnesium ammonium phosphate and cystine [4]. Urolithiasis is often detected accidentally during an ultrasound or an abdominal x-ray performed for other reasons. If patients experience any problems connected with the presence or passage of stones in the urinary tract, they usually present with a colic pain located in the flank, called renal colic. It is usually a pain of sudden onset and in half of the cases it is associated with nausea or vomiting. Severe pain can lead to hypotension and fainting [5]. Unlike biliary colic or intestinal colic, the pain is often continuous. Any periods of aggravation or reduction of the ailment result from the spontaneous passage of ureteral calculi [6]. The exact location and radiation of the pain depends on the location of the stone in the urinary tract. It has not been explicitly established so far whether stones in the pyelocalyceal system can cause any pain if they do not block the urinary flow. In cases where the stone is in the ureteropelvic junction, the patient’s pain is located in the costovertebral angle on the side of the deposit. The pain may then radiate along the ureter, but this is more characteristic for stones which are located lower. In addition, patients may report dysuria, suprapubic pain, frequent micturition. A stone located in the ureter can cause severe, acute pain radiating to the groin, testicles in males or labia majora in females, especially if it is situated in the distal part of the ureter. Stones located in the middle of the ureter manifest by acute pain radiating to the lower quadrant of the abdominal cavity. Stones that got into the bladder usually do not cause any pain [6,7]. In the physical examination we usually find a positive renal Murphy’s punch sign on the side of the stone (severe pain after percussion of the area of the costovertebral angle). Intestinal motility may be decreased. Patients with renal colic do not present with any peritoneal signs. Unlike patients with acute surgical conditions, these patients are usually agitated, restless, looking for a position of the body that minimizes pain. In boys, the palpation of the testicle may be painful, but it is not inflamed. Moreover, patients with renal colic usually have tachycardia and high blood pressure [6, 7]. It should be emphasized that typical renal colic symptoms occur in children over the age of 10. In younger patients the symptoms are often very uncommon, such as loss of appetite, diarrhea, vomiting, anxiety, unexplained fever. In some patients, dysuria or hematuria may be the only symptoms. We should also remember about the possibility of urolithiasis in case of recurrent vulvitis or balanoposthitis. The passage of small deposits may irritate these structures [8]. Additional tests must be performed to confirm the clinical diagnosis of renal colic. Microscopic hematuria can be found in routine urinalysis. The first most commonly performed study is abdominal ultrasound with estimation of the deposit size and evaluation of urinary tract obstruction. An alternative or complementary study is an abdominal x-ray, where radiopaque deposits can be shown. The gold standard is intravenous urography, which is currently being displaced by the unenhanced helical computed tomography of the abdomen [7]. Differential diagnosis should include: acute pyelonephritis, biliary colic, “acute abdomen”, aortic aneurysm and other causes of urinary tract obstruction caused by blood clots or necrotic kidney tissue in acute renal papillary necrosis [5, 7].

## Pathophysiology of pain in renal colic

The pain caused by urinary stones is usually due to two reasons. The first is physical stretching of the collecting system or the ureter as a result of a sudden blockage that inhibits the flow of urine through the urinary tract. The second one is stretching of the kidney fibrous capsule. The type of pain in a particular patient is difficult to distinguish, usually both of them overlap [7]. Mostly in the first case the pain is probably similar to a colic [6]. There are pain receptors in the submucosa of the pyelocalyceal system and the upper part of the ureter which are stimulated by the excessive dilatation of these structures. Contractions of urinary tract muscles, increased peristalsis or stimulation of the urinary tract chemoreceptors by the deposit’s passage play a significantly reduced role. Pain in the renal colic is visceral. Impulses from the pain receptors are transmitted by sympathetic fibers through the aorticorenal ganglion, celiac plexus and superior mesenteric ganglion. Processing nociceptive information (pain information conducted by electrical pulse) from these parts of the urinary tract takes place at the level of Th11 to L2 in the spinal cord and then the pulse mainly moves through the spinothalamic tract. The severity of pain depends on the individual pain threshold and on the change in hydrostatic pressure in the part of the urinary system above the obstruction. It does not depend on the size of the deposit. The highest hydrostatic pressure is observed 2 to 5 hours after the blockage of urinary flow. Prolonged deposition of the stone in one place causes the activation of autoregulatory mechanisms to lower the pressure of the upper urinary tract, which limits the pain. Initially, blood flow through the kidney is increased as a result of dilation of the afferent renal arterioles and increased hydrostatic pressure, but after about 5 hours these parameters begin to decrease. Along with reduced blood flow, urine production (glomerular filtration) also decreases. After 3 days the blood flow through the kidney is reduced by 50% and within 8 weeks it decreases to 12% of the initial value. Then the pressure in the urinary tract above the obstruction is close to normal. At the same time blood flow in the second kidney is increased to maintain normal kidney function. In addition, in case of a prolonged blockage of the urine flow in the affected kidney interstitial oedema occurs, which in turn increases lymphatic drainage of this area. After the initial increase of ureteral contractions, it then results in a significant reduction of ureteral peristalsis. Dilatation of the ureter in front of the blockage to a certain extent allows the urine to flow to the distal part of the urinary tract. All of these mechanisms are aimed at establishing a new balance that keeps kidney function normal. They also allow to explain why in most patients pain passes after about 24 hours, despite persistent blockage of urine flow. In case when partial urine flow is possible next to the stone, similar mechanisms occur, however with less intensity and slower dynamics [6].

## Treatment of renal colic

The goal of renal colic management is to reduce pain and increase the possibility of spontaneous stone passage. A significant group of the affected patients can be treated on outpatient basis. The condition to enroll the patient in ambulatory care is a good general state and the size of the stone under 5mm. In this case deposits are more likely to pass spontaneously. Hospital admission of a patient with renal colic is indicated if the pain is not relieved by initial treatment, there is vomiting with a risk of dehydration, the patient has only one kidney or uncontrolled diabetes [9]. If there is a fever it requires an absolute exclusion of urinary tract infection, because its coincidence with urinary obstruction increases the risk of complications [6]. In case of qualifying the patient for outpatient treatment, stone passage should be monitored periodically by ultrasound or X-ray [9]. The basic treatment for renal colic is analgetic therapy. The most commonly used drugs are NSAIDs and opiates. Elevated upper urinary tract pressure due to the presence of stones stimulates prostaglandin production. They cause ureteral contractions, higher diuresis levels, inflammatory reaction and oedema. Therefore, NSAIDs besides analgesics reduce oedema in the area of the stone, which may facilitate its passage [8]. In case of a minor aggravation of pain, the oral or rectal route of administration is sufficient. Initially we usually use ketoprofen, ibuprofen, diclofenac, naproxen. Another option is a fixed combination containing paracetamol and codeine [5]. Some of these have been registered in patients over 12 years old. In case of severe pain, intravenous or intramuscular drug administration is preferred. Metamizol is often used in the pediatric population. Opioid drugs used for kidney colic pain include pethidine, tramadol, morphine sulphate. It has been shown that the use of NSAIDs in patients with renal colic is superior to monotherapy with opioids, which should be used mainly in combination therapy [7]. The effectiveness of desmopressin, which is antidiuretic and inhibits renal pelvic contractions, has also been proven. It may be complementary to NSAID therapy providing that the intake of fluuids is monitored precisely [8]. Another important component of renal colic treatment are medications that facilitate urinary stone passage by reducing oedema (as mentioned above, NSAIDs or corticosteroids) or limiting urethral contractions. The direct cause of ureteral contraction is increased cytoplasmic calcium concentration. Calcium channel blockers act by blocking the influx of calcium ions into vascular smooth muscle and that is why the ureteral wall relaxes.

There are alpha adrenergic receptors in the ureter which when stimulated promote smooth muscle contraction. Therefore, there is an indication for the use of alpha blockers during colic pain. Relaxation of ureteral smooth muscle can also be achieved by inhibiting the activity of the phosphodiesterase enzyme (papaverine and its derivatives e.g. drotaverine work this way). It would theoretically be expected to reduce the symptoms of renal colic during antimuscarinic therapy but no effectiveness of N-butylscopolamine in reducing pain or facilitating stone passage has been shown [7]. Intensive hydration is not currently recommended. Patients who are unlikely to spontaneously excrete the stone are eligible for minimally invasive treatment. There are three possible approaches that are applied: extracorporeal shock wave lithotripsy (ESWL), ureteroscopic lithotripsy (URL) or percutaneous nephrolithotripsy (PCNL). In occasional cases surgical treatment is necessary [8].

2 to 3 months after the first attack of renal colic, when the stone passage was successful, initial diagnostic tests should be performed: routine urinalysis, serum creatinine, sodium, potassium, phosphorus and uric acid levels and arterial blood gas analysis. It is recommended abdominal ultrasound should be performed one year after renal colic and then every 2 years. Additional metabolic tests including urinary calcium, phosphate, uric acid, citrate, oxalate, creatinine and cystine excretion are recommended in all children, in patients with multiple stones, with a high activity of stone formation, with one kidney, with kidney failure. If the stone is filtered out of the urine analysis of its chemical composition should be performed [10].

## Prognosis

It is estimated that about 80% of ureteric stones, mainly under 5 mm, pass spontaneously. Spontaneous passage of larger stones was also observed, especially in children, due to their more elastic urinary tract. An adverse prognostic factor for spontaneous stone passage occurs when it remains in one place (>4 weeks) [8]. Animal studies have shown that irreversible changes in renal function can occur after 5-14 days of complete blockage of urine flow. Therefore, it is not recommended to perform conservative treatment for too long if the stone passage is not observed [6]. A dangerous complication of urolithiasis is also urinary tract infection that can lead to urosepsis. Urolithiasis has a high recurrence risk, reaching up to 40% in 5-year follow-up and up to 50% in 10 years. According to other authors, it may be even higher [10].

To illustrate the above theoretical statements, we present a case report concerning a boy with multiple episodes of renal colic.

A boy with thrombocytopenia and absent radius syndrome (TAR syndrome) was admitted for the first time to the Department of Paediatrics, Nephrology and Allergology, Military Institute of Medicine at the age of 14 months because of urolithiasis, to perform the ESWL procedure.

Urolithiasis was identified at the age of 6 months during diagnostics of erythrocyturia. Before the boy was admitted to our department, he had been diagnosed in a different health center, where hypercalciuria was detected. The cystouretrography that was taken at that time showed posterior urethral valves. Additionally, in the urography there was dilatation of the right kidney pelvis with filling defects consistent with kidney stones. The child had never had a urinary tract infection. Urethral valve ablation was performed.

The boy presented at the department with numerous deposits in the right kidney calices and pelvis. One of them was located in the ureteroplevic junction and caused urostasis in the pyelocalyceal system. The boy was eligible for Extracorporeal Shock Wave Lithotripsy (ESWL). The ultrasound taken after the treatment showed disintegration of deposits and reduction of urinary stasis.

The next ESWL, due to the deposits in inferior calices in the right kidney, was performed at the age of three. Ultrasonography showed disintegration of the deposits and no retention in this kidney. The boy passed the stones.

Since then, the patient had not attended any nephrological control for almost 2 years.

At the age of 4 the boy returned to the clinic because of abdominal pain. The ultrasound revealed deposits in the right kidney pelvis and inferior calices. The third ESWL treatment in his life, with antibiotic prophylaxis, was performed, after which disintegration of the deposits was found in the ultrasound study. The boy passed the stones again.

Three weeks after the ESWL procedure the boy was again admitted to the clinic with severe abdominal pain and vomiting. Ultrasound showed deposits in the right kidney inferior calices and pelvis and in ureterovesical junction; urinary flow was normal. Intravenous fluids with spasmolytics (drotaverine 40mg intravenously), furazidin (50mg per os) and analgetics (paracetamol 270mg, tramadol 30mg, metamizole 5g intravenously) were used in the treatment. The symptoms resolved and the stone was spontaneously passed. The urological consultant recommended URSL. However, due to the spontaneous excretion of the deposits, the procedure was abandoned.

After another three weeks, the symptoms of renal colic reappeared. Abdominal ultrasound showed a dilated pyelocalyceal system of the right kidney and stones in the pelvis and inferior calices of this kidney. The fourth ESWL procedure was performed with antibiotic prophylaxis and under general anaesthesia. The boy excreted deposits after the treatment. Due to severe abdominal pain, he was given spasmolytics (drotaverine 40mg intravenously), analgetics (paracetamol 270mg, tramadol 30mg, metamizole 5g intravenously) and doxazosinum (1mg per os). The following ultrasound scans showed a slightly extended collecting system and two small deposits in his inferior calices.

The boy was hospitalized in the clinic with renal colic two more times, 3 weeks and 1.5 months after the last ESWL procedure. Spasmolytics (drotaverine) and analgetics (paracetamol and tramadol) were used in the treatment. Due to recurrent renal colic attacks and their severe nature, it was decided to perform abdominal computed tomography urography. The study showed a dilated pyelocalyceal system and a 5mm stone in the inferior calices in the right kidney. The lack of indications contributed to giving up another ESWL procedure. Idiopathic hypercalciuria was diagnosed as the reason for multiple episodes of renal colic. Since then, no further episodes of renal colic have been noticed.

## Summary

Symptoms of renal colic are usually observed in older children (over 10 years of age) but this case proves that they may also occur in younger ones. The conservative treatment that was administered, including the use of alpha-blockers, allowed to manage the pain and to result in effective stone passage. This seems to be an indication for the use of alpha-blockers early on in the treatment, when the crushed stone fragments pass to the ureter.
